# Context, Culture, and the Complexity of De-Implementing Low-Value Care: Comment on "Key Factors that Promote Low-Value Care: Views of Experts From the United States, Canada, and the Netherlands"

**DOI:** 10.34172/ijhpm.2022.6968

**Published:** 2022-02-09

**Authors:** Emma E. Sypes, Jeanna Parsons Leigh, Henry T. Stelfox, Daniel J. Niven

**Affiliations:** ^1^Faculty of Medicine, University of Ottawa, Ottawa, ON, Canada.; ^2^School of Health Administration, Faculty of Health, Dalhousie University, Halifax, NS, Canada.; ^3^Department of Critical Care Medicine, University of Calgary and Alberta Health Services, Calgary, AB, Canada.; ^4^Department of Community Health Sciences, University of Calgary, Calgary, AB, Canada.; ^5^O’Brien Institute for Public Health, University of Calgary, Calgary, AB, Canada.

**Keywords:** Low-Value Care, Overuse, De-Implementation

## Abstract

Low-value care contributes to poor quality of care and wasteful spending in healthcare systems. In Verkerk and colleagues’ recent qualitative study, interviews with low-value care experts from Canada, the United States, and the Netherlands identified a broad range of nationally relevant social, system, and knowledge factors that promote ongoing use of low-value care. These factors highlight the complexity of the problem that is persistent use of low-value care and how it is heavily influenced by public and medical culture as well as healthcare system features. This commentary discusses how these findings integrate within current low-value care and de-implementation literature and uses specific low-value care examples to highlight the importance of considering context, culture, and clinical setting when considering how to apply these factors to future de-implementation initiatives.

 De-implementing low-value care is a major challenge within healthcare systems around the world.^1^ The perpetuated use of healthcare services that provide little or no benefit to patients, or which may cause harm, represents wasteful consumption of healthcare resources.^2^ Since the launch of the Choosing Wisely Campaign in 2012, there has been an exponential increase in research identifying hundreds of low-value practices across all areas of healthcare.^3-5^ Although many low-value practices have been identified as candidates for de-implementation, their use persists because the process of changing engrained clinical behaviour is complex. While we have established theories, models, and frameworks to guide the process of implementing high-value care into practice, less is known about the process of de-implementing low-value care. Studies have begun to further unravel the complex interplay between processes and determinants (ie, barriers and facilitators) of de-implementation and implementation. Nevertheless, despite advancements in our understanding of de-implementation, low-value care remains a major burden within healthcare systems throughout the world.

 Prior to the coronavirus disease 2019 (COVID-19) pandemic, reducing low-value care was increasingly recognized as a priority for healthcare system improvement. Now, owing to the many negative health system impacts of COVID-19 (eg, delayed diagnoses and treatments), reducing low-value care should be an even greater priority.^6,7^ Ensuring that healthcare providers are delivering high-value care will help mitigate the resource and financial constraints that will impact healthcare systems post-pandemic.^8^ In Verkerk and colleagues’ recent study “Key factors that promote low-value care: views of experts from the United States, Canada, and the Netherlands,”^9^ the authors aimed to explore the factors that promote ongoing use of practices identified as low-value. This commentary will review the article by Verkerk et al, highlight key findings, and offer further consideration for how their findings may be interpreted and applied to future initiatives to reduce low-value care.

 Verkerk et al interviewed 18 experts from Canada, the United States, and Denmark. Pre-existing frameworks describing drivers of poor medical care and determinants of healthcare professional practice were used to guide interviews and elicit factors that promote low-value care. This enabled the authors to fill a gap within the literature and potentially identify social and system-level factors that are often overlooked, yet at a macro-level are potentially very influential.^10^ Key factors promoting use of low-value care that emerged from the interviews included social factors (public and medical culture), system factors (payment structure, influence from industry, malpractice litigation), and knowledge factors (evidence, medical education). The identification and description of these factors are a meaningful addition to the body of literature describing determinants of low-value care^11,12^ and offer potential strategies to reduce its overuse.

 Generalizability of many of the key factors promoting low-value care likely depends on context. For example, one of the social factors promoting low-value care identified in the study by Verkerk et al was public culture, and the tendency to believe that ‘more is more.’ This suggests that patients may value the receipt of tests and treatments because it makes them feel like something is being done to help them, and understanding this, clinicians may decide to provide tests or treatments when the clinical indication may be weak or absent. Patient education materials, such as those from the Choosing Wisely campaign, have been shown to increase patients’ awareness of low-value care and encourage them to initiate conversations about the value of their care with their physicians.^13^ The importance of patient perceptions likely varies across clinical contexts. Diagnostic imaging for low-risk low back pain is an example of a low-value practice where patients’ expectations or preferences have been shown to significantly influence utilization.^14^ Healthcare providers have reported that more patient education and additional time to explain their rationale to a patient would help them reduce low-value imaging for low back pain.^15^ Targeting patient expectations through implementation of an intervention within the patient-clinician interaction in primary care may provide an opportunity for the patient to express their preferences and engage in a discussion about the merits of imaging. A national intervention in Australia applied this approach to the patient-clinician interaction regarding imaging for low-back pain. In their study, patient-specific educational tools and clinician-targeted decision-support tools were implemented to assist with decision making regarding imaging for low-back pain.^16^ They found that this intervention reduced primary care ordering of imaging by nearly 11% over the study period. Similar results have been achieved with interventions targeting the patient-clinician interaction in other primary care contexts, such as with antibiotic prescribing for upper respiratory tract infections and diagnostic imaging for low-risk head injuries.^17^

 In contrast to primary care, where decisions regarding use of low-value tests or treatments are commonly made during the patient-clinician interaction, acute care, and in particular the intensive care unit (ICU), is a care environment where some of the decisions regarding care required to save life or limb may be less influenced by public culture. For example, several studies suggest that for most patients admitted to adult ICUs, a hemoglobin target of 7 g/dL is sufficient, and transfusion to higher hemoglobin levels that more closely resemble normal values is associated with worse outcomes.^18^ Red blood cell transfusion when the hemoglobin is 7 g/dL or higher is, for most patients, low-value care. Owing to their severe illness, ICU patients are not aware that their hemoglobin level may be lower than normal, whereas the clinicians are, and thus best positioned to make decisions regarding the merits of transfusion. In this case, an intervention that targeted patients or their family members would be less impactful than one focusing more heavily on clinicians, their medical knowledge, and the strong medical culture that more care and normalization of physiology is better.

 The more care is better culture and the ability of clinicians to adapt established medical practice patterns in response to new evidence are major barriers to reducing use of low-value care that likely transcend all areas of medicine. It is hard for clinicians to unlearn patterns of practice that have emerged from years of medical training and experience.^19-21^ A recent qualitative evidence synthesis indicates that clinician knowledge is a commonly reported determinant of low-value care,^12^ yet it is less clear how this should be addressed. Clinicians engage with multiple sources of evidence (eg, journal articles, clinical guidelines) within a medical culture with established norms whilst also subject to their own cognitive biases. All of these elements may contribute to how they interact with and apply their medical knowledge surrounding low-value care.^22^ Clinicians are also faced with patients whose complexity frequently exceeds that of those examined in clinical trials, and therefore have difficulty applying evidence to the clinical contexts they encounter. Additional work is required to further explore with clinicians their own experiences interacting with new potentially contradictory evidence and the decision to de-implement care that may no longer be considered high value.

 In addition to social and knowledge factors, the system in which care is delivered has been shown to influence the delivery of low-value care. For example, a study examining vitamin D screening in the United States and Canada found modest reductions in low-value screening following the release of Choosing Wisely recommendations.^23^ However, when a new payment policy eliminating reimbursement for the screening was introduced in Ontario, Canada, the rate of screening was reduced by 93%.^23^ Here, an intervention addressing system-level factors was needed in addition to the Choosing Wisely Campaign, which targets knowledge and social factors. Differences in the structure of healthcare systems suggests that context specific interventions may need to be considered. A systematic review of interventions to reduce low-value care identified the importance of system-level strategies that aimed to reduce demand of low-value care (eg, patient cost-sharing that incentivizes high-value care over low-value care) and supply of low-value care (eg, value-based pay-for-performance).^24^ Research suggests that effective interventions that reduce low-value care are more commonly multi-component interventions that address both system-level factors (eg, payment structure, policy changes) and social and knowledge factors.^24^

 The factors identified by Verkerk et al complement those cited within the current low-value care and de-implementation literature. Two recent evidence syntheses of determinants of low-value care suggest patient and provider characteristics (eg, knowledge, attitude, behaviours) to be the most cited determinants of low-value care.^11,12^ Other factors outside the patient-provider dynamic like the system-level factors identified by Verkerk et al appear to be less commonly reported in the literature, but as demonstrated by Verkerk’s findings, this does not dismiss their impact on low-value care. Verkerk’s study is an important reminder that no single determinant is responsible for the challenges associated with reducing low-value care; social, knowledge, and system-level factors are driving low-value care in an interconnected manner. When designing de-implementation interventions, these social, knowledge, and system factors should be evaluated to understand what the predominant driver of use of the specific low-value practice is and what might work best to reduce its use. As highlighted in this commentary, these factors are likely going to look different depending on the target low-value practice, care setting and health system.

 In conclusion, the study by Verkerk et al highlights key social, knowledge, and system factors that promote low-value care and underscores the complexity of the challenge of de-implementation. Understanding how these key factors vary with contextual factors such as the specific low-value practice and clinical setting is an important consideration in the design of de-implementation interventions. It is essential that we engage all relevant stakeholders, including clinicians and patients, as we continue to build the body of evidence describing determinants of low-value care, pursue initiatives to reduce low-value care, and advance the science of de-implementation.

## Ethical issues

 Not applicable.

## Competing interests

 Authors declare that they have no competing interests.

## Authors’ contributions

 Conception and design: EES, JPL, HTS, and DJN. Drafting of the manuscript: EES, JPL, HTS, and DJN. Critical revision of the manuscript for important intellectual content: EES, JPL, HTS, and DJN. Supervision: EES, JPL, HTS, and DJN.
